# Full-Mouth Rehabilitation of a Patient With Generalized Enamel Hypoplasia and an Anterior Open Bite Using a Physiologic Approach: A Case Report

**DOI:** 10.7759/cureus.110576

**Published:** 2026-06-10

**Authors:** Priyanka A Patil, Pronob K Sanyal, Aaditee V Vande

**Affiliations:** 1 Department of Prosthodontics, School of Dental Sciences, Krishna Vishwa Vidyapeeth (Deemed to Be University), Karad, IND

**Keywords:** anterior open bite, enamel hypoplasia, full-mouth rehabilitation, lucia jig, porcelain-fused-to-metal restorations, shimbashi number, swallowing method, vertical dimension of occlusion

## Abstract

Enamel hypoplasia is a developmental defect characterized by quantitative enamel deficiency, often leading to esthetic compromise, hypersensitivity, occlusal disharmony, and loss of vertical dimension. Rehabilitation of such patients is clinically demanding, particularly when associated with an anterior open bite. This case report describes the prosthodontic management of a 44-year-old male patient with generalized enamel hypoplasia and an anterior open bite through systematic full-mouth rehabilitation. A physiologic approach using the swallowing method was employed to determine the vertical dimension of occlusion, followed by staged rehabilitation with porcelain-fused-to-metal restorations. The treatment resulted in improved function, esthetics, and occlusal stability with satisfactory patient acceptance. This report emphasizes the importance of physiologic vertical dimension determination and structured prosthodontic planning in complex rehabilitative cases.

## Introduction

Enamel hypoplasia is a developmental disturbance that occurs during enamel matrix formation, resulting in quantitatively deficient enamel. Enamel hypoplasia may be classified according to severity as mild, characterized by single or multiple shallow or deep pits representing localized enamel loss; moderate, characterized by grooves or linear defects of enamel loss measuring less than 2 mm in width; and severe, characterized by partial or complete absence of enamel involving the crown of the affected tooth. Clinically, it presents as thin, pitted, or irregular enamel surfaces and is frequently associated with dentinal hypersensitivity, accelerated tooth wear, compromised esthetics, and occlusal discrepancies. In severe generalized cases, progressive enamel loss may adversely affect mastication, phonetics, and the vertical dimension of occlusion (VDO), often necessitating comprehensive prosthodontic rehabilitation [1,2].

Patients with generalized enamel hypoplasia commonly present with functional and esthetic challenges that significantly affect oral health-related quality of life. Management of such cases becomes more complex when associated with an anterior open bite and altered occlusal vertical dimension. Successful rehabilitation requires careful integration of biologic, functional, and esthetic principles to restore both oral function and facial harmony.

The VDO is defined as the facial height measured when the maxillary and mandibular teeth, occlusal rims, or other occluding stops are in contact with the mandible in centric relation or centric occlusion. In contrast, the mandibular rest position represents the physiologic, relaxed position of the mandible when the head is upright and the muscles of mastication are minimally contracted. Accurate determination of VDO is a critical step during full-mouth rehabilitation, as inappropriate alteration may result in muscle fatigue, temporomandibular discomfort, compromised phonetics, and prosthetic failure. Various mechanical, phonetic, esthetic, and physiologic methods have been proposed for determining VDO. Among these, physiologic techniques, particularly the swallowing method, are considered reliable because mandibular positioning during swallowing is governed by neuromuscular coordination and represents a repeatable functional position. The functional act of swallowing plays an important role in establishing a reproducible mandibular position and occlusal relationship [3].

The repetitive contracted length (RCL) of the elevator muscles is considered an important physiologic determinant in establishing a stable and functional VDO. The vertical dimension is primarily governed by the RCL of these muscles, which controls the postural closure of the mandible and determines the point at which occlusal contact occurs and adaptive forces are neutralized. Consequently, the consistent contraction of the elevator muscles helps maintain a specific and stable VDO that is harmoniously adapted to the occlusal plane. The constant functional pattern of swallowing saliva serves as a physiologic basis for establishing mandibular position and occlusion [3].

Although several treatment modalities for full-mouth rehabilitation have been described in the literature, limited clinical reports have documented the application of physiologic VDO determination in patients with generalized enamel hypoplasia associated with an anterior open bite. Therefore, this case report describes a systematic prosthodontic approach for the rehabilitation of such a patient, with emphasis on physiologic determination of VDO using the swallowing method.

## Case presentation

**Figure 1:**
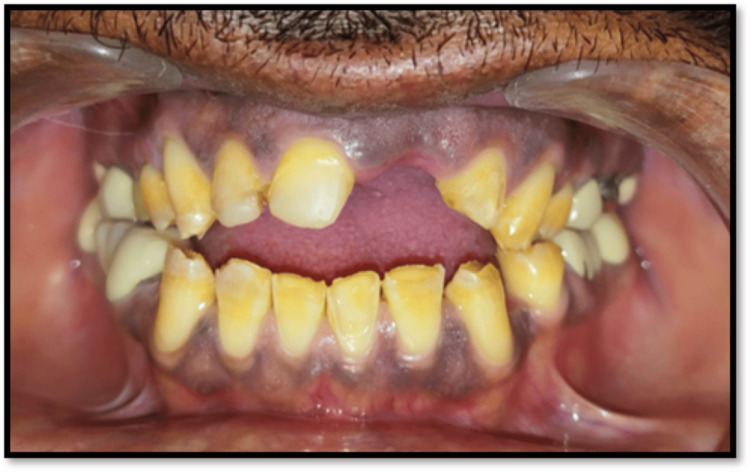
Intraoral frontal view showing generalized yellow discoloration of teeth with partial enamel loss involving labial, occlusal, and incisal surfaces. An anterior open bite is evident, along with a missing maxillary left central incisor (21).

**Figure 2:**
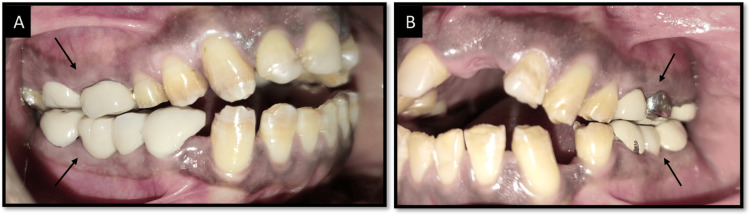
Intraoral right-side view (A) showing existing prosthetic restorations, including porcelain-fused-to-metal (PFM) crowns on teeth 16 and 17, and PFM bridge 44–47 left side view and (B) showing PFM crowns 25, 26, and 27, and PFM bridge involving 35–37. Porcelain fractures are evident in teeth 26 and 35. A unilateral crossbite is present on the right side, along with Angle’s Class II molar relationship on the left side

**Figure 3:**
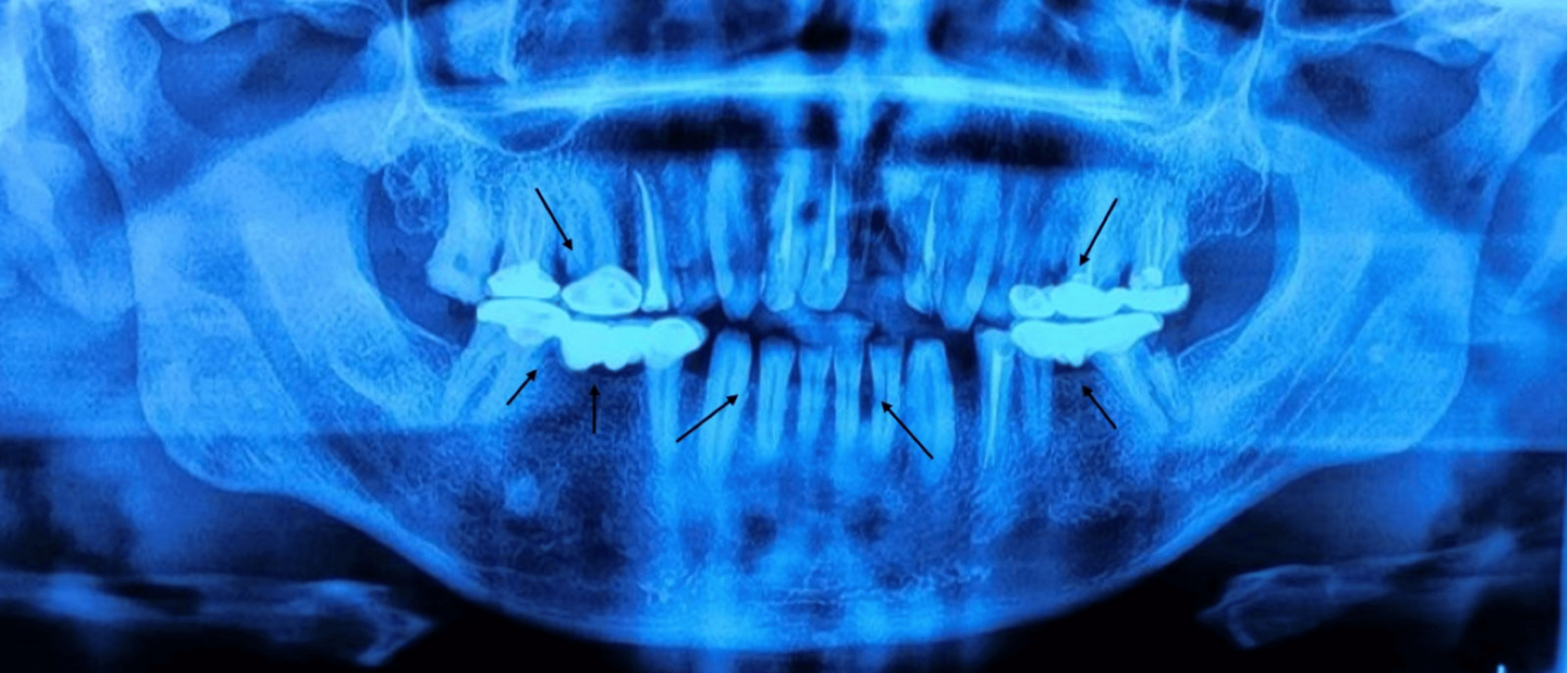
Panoramic radiograph demonstrating generalized enamel deficiency affecting multiple teeth, with existing prosthetic restorations visible. No significant periodontal involvement or bone loss is observed

A 44-year-old male patient presented to the Department of Prosthodontics with complaints of compromised esthetics, difficulty in mastication, and sensitivity in multiple teeth. Clinical examination revealed generalized enamel hypoplasia characterized by severe enamel loss, yellow discoloration, fragile teeth, and occasional chipping noted since early childhood affecting multiple teeth, along with an anterior open bite (Figure 1). Additionally, a unilateral crossbite was present on the right side, and Angle’s Class II molar relationship was observed on the left side. Tooth 21 was missing (Figure 1). Existing prosthetic restorations included porcelain-fused-to-metal (PFM) crowns on teeth 16, 17, 25, 26, and 27, and PFM bridges involving 35-37 and 44-47. Endodontic treatment had been performed on teeth 11, 12, 15, 22, and 34. Porcelain fractures were noted in teeth 26 and 35 (Figure 2). Radiographic evaluation confirmed generalized enamel deficiency without significant periodontal involvement (Figure 3).

Following informed consent, a comprehensive full-mouth rehabilitation was planned with the aim of restoring function, esthetics, and occlusal stability.

**Figure 4:**
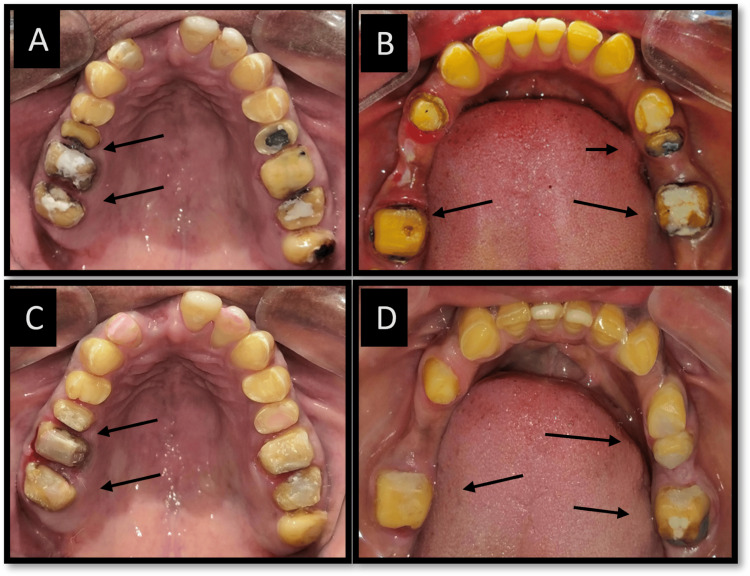
Intraoral pre-operative occlusal views (A and B) showing multiple carious lesions and defective restorations in the maxillary and mandibular arches. Post-operative views (C and D) following caries excavation, with completed restorative procedures and core build-ups to reinforce the tooth structure and provide adequate support for subsequent prosthetic rehabilitation

All existing prostheses were carefully removed to allow accurate assessment of the underlying tooth structure and occlusal relationships. After removal, the teeth were evaluated for caries, marginal discrepancies, and structural integrity. Carious lesions were excavated, and necessary restorative procedures and core build-ups were performed to reinforce the tooth structure and provide adequate support for future restorations (Figures 4A-4D).

**Figure 5:**
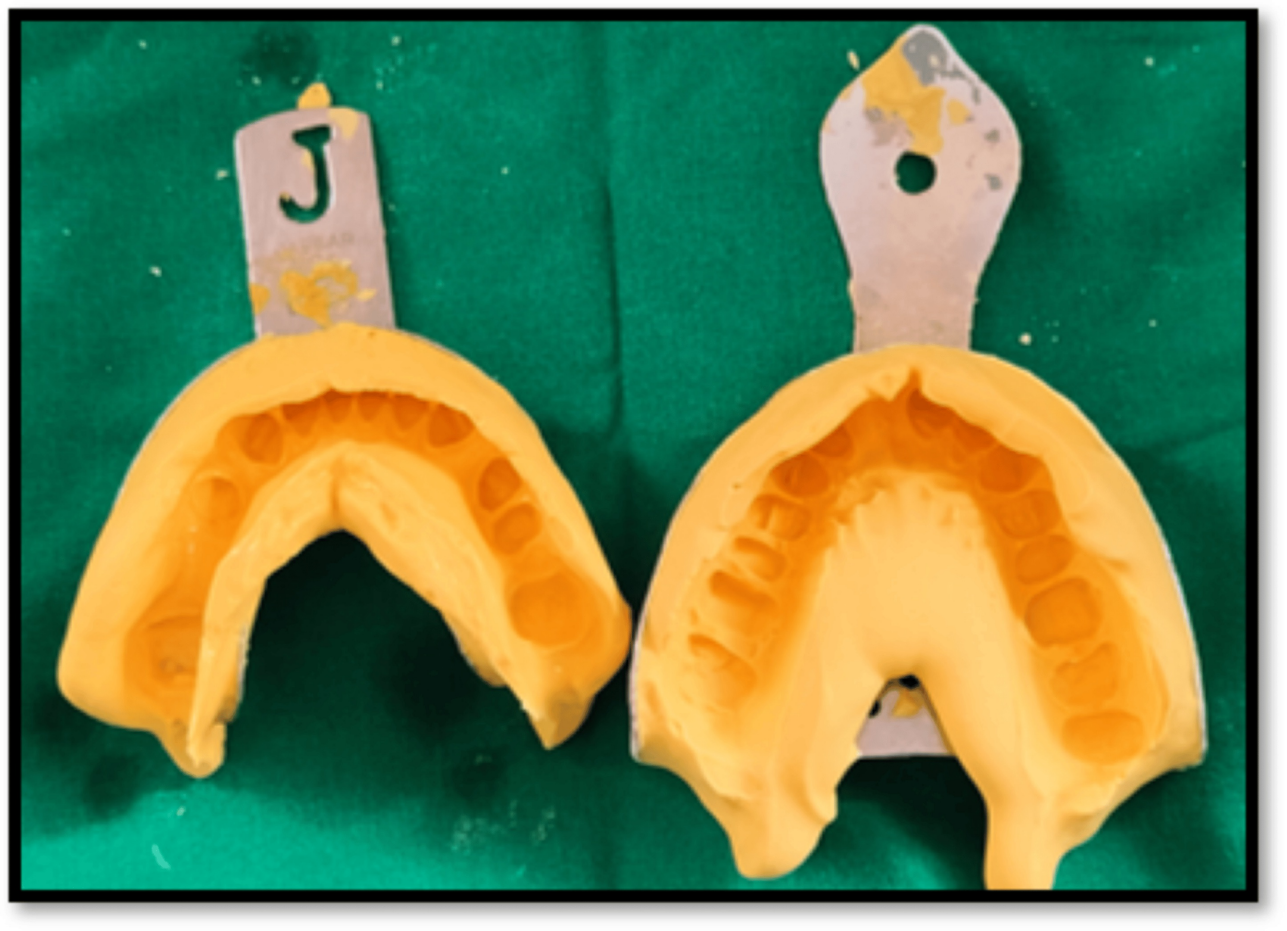
Diagnostic impression of the dental arches made using an irreversible hydrocolloid material for preliminary cast fabrication and treatment planning

**Figure 6:**
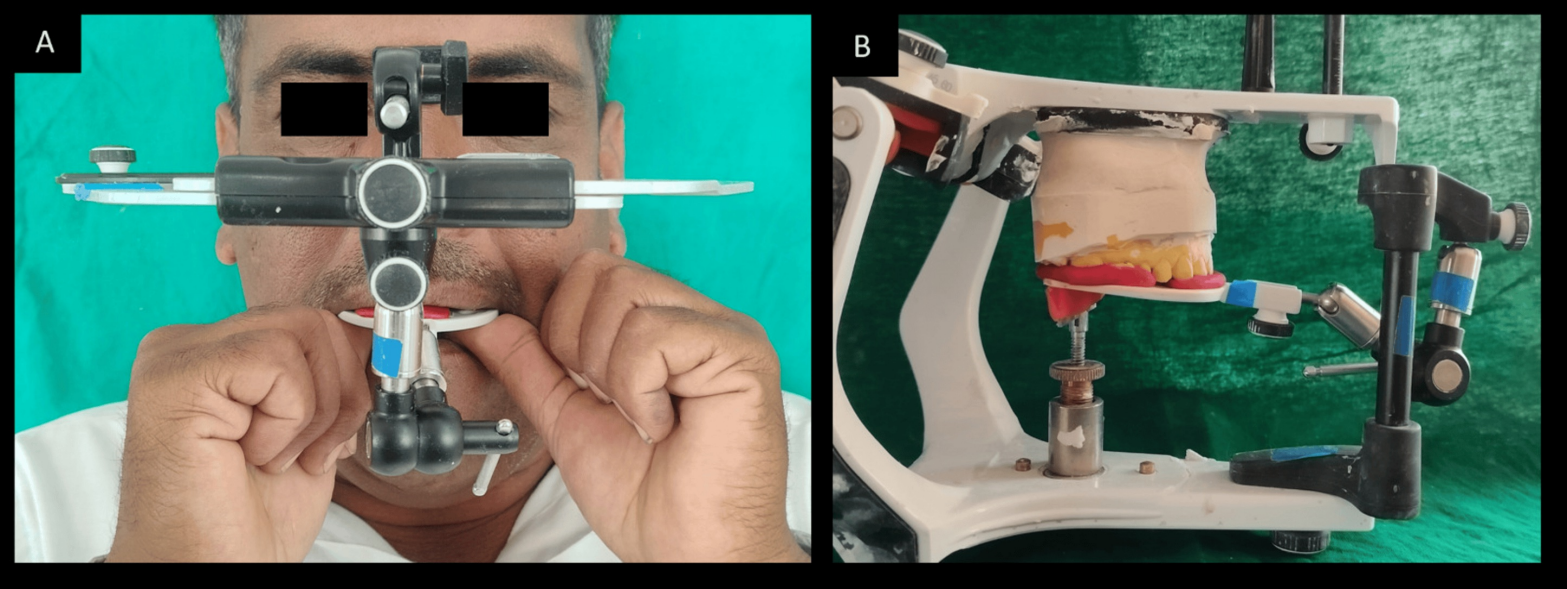
Mounted diagnostic casts on a semi-adjustable articulator using a facebow transfer

Diagnostic impressions were made using irreversible hydrocolloid (Figure 5), and the resulting casts were mounted on a semi-adjustable articulator using a facebow transfer to accurately relate the maxillary arch to the temporomandibular joint (Figure 6).

**Figure 7:**
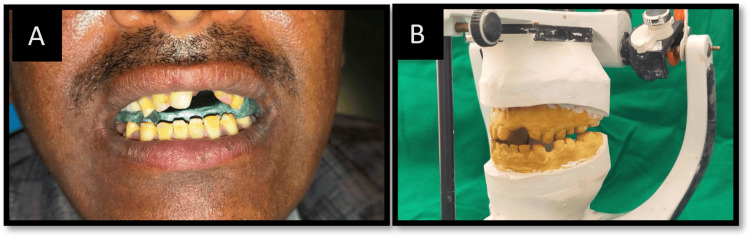
(A) Determination of vertical dimension of occlusion using the physiologic swallowing method. (B) Mounting of the mandibular cast at the established vertical dimension

The VDO was determined using the physiologic swallowing method. The patient was instructed to swallow repeatedly, and the mandibular position during swallowing was recorded, as it represents a reproducible and neuromuscularly balanced position, and the mandibular cast was mounted (Figures 7A, 7B).

**Figure 8:**
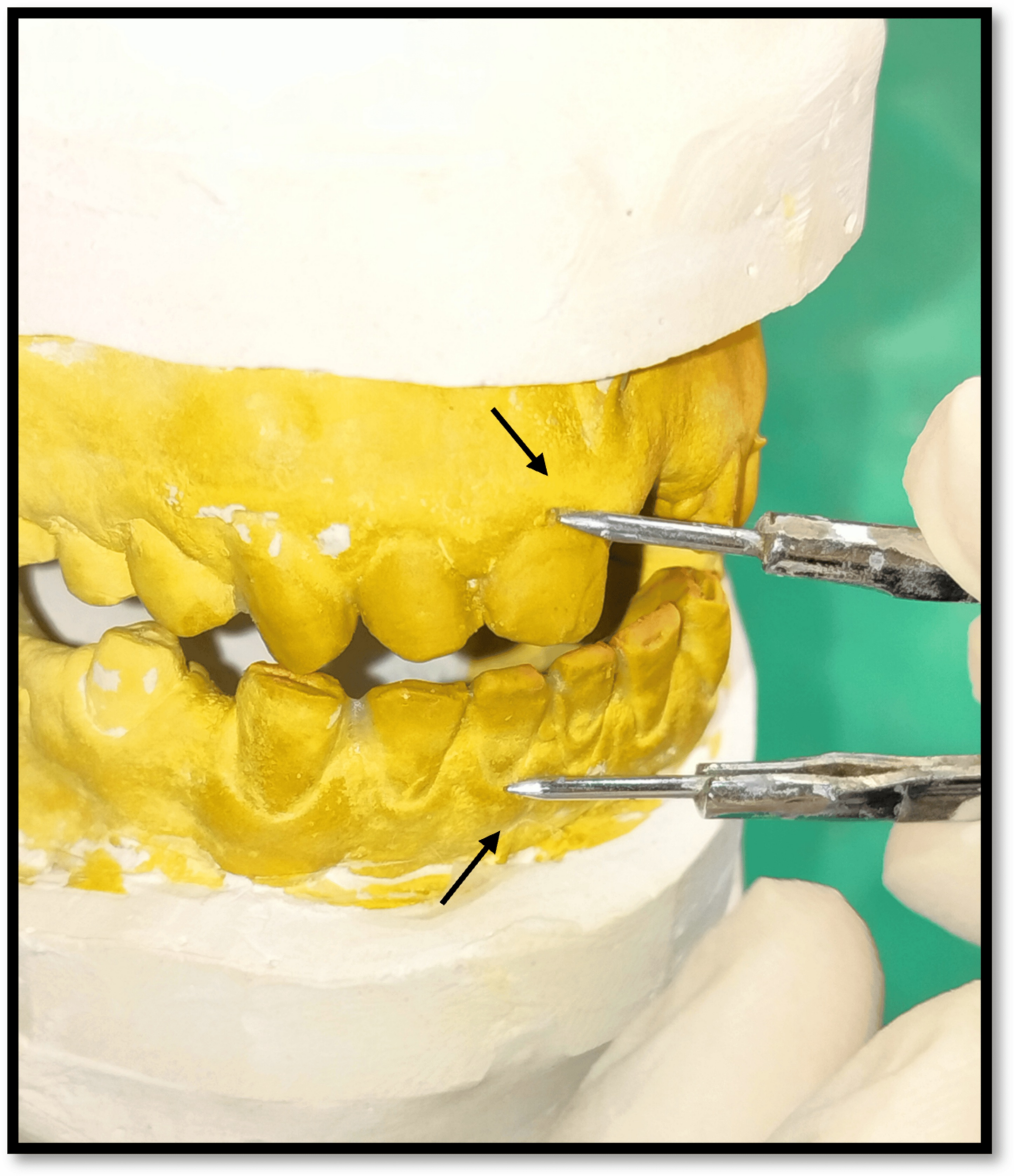
Measurement of the Shimbashi value performed to verify the established vertical dimension of occlusion during treatment planning

**Figure 9:**
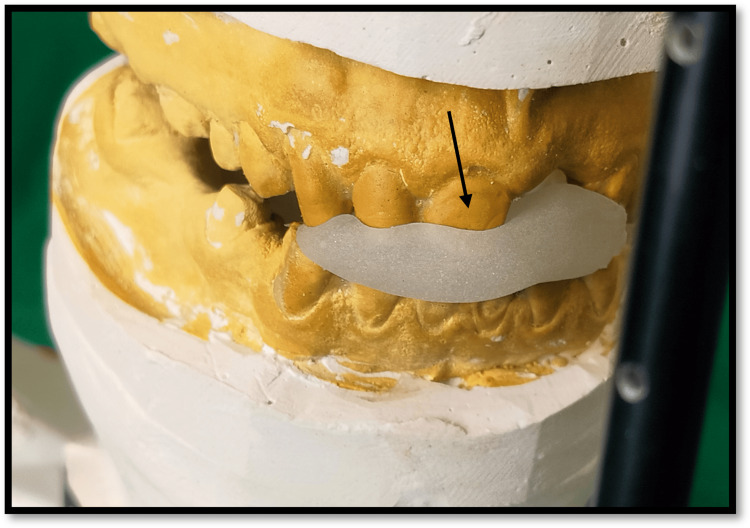
Intraoral view showing the fabricated Lucia jig used to maintain centric relation and vertical dimension of occlusion

To further verify the vertical dimension, the Shimbashi value was measured (Figure 8). A Lucia jig was fabricated to maintain centric relation and vertical dimension (Figure 9). A diagnostic wax-up was subsequently performed to visualize the planned prosthetic outcome.

**Figure 10:**
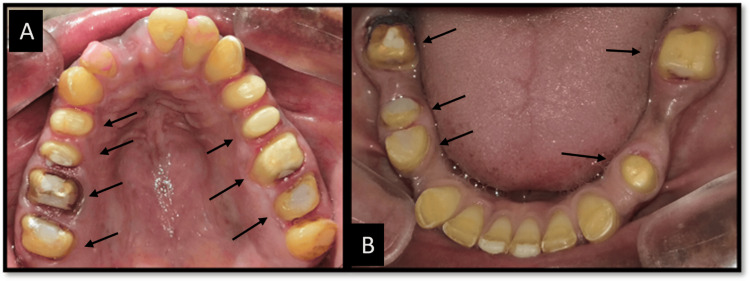
Intraoral occlusal view showing preparation of posterior teeth

**Figure 11:**
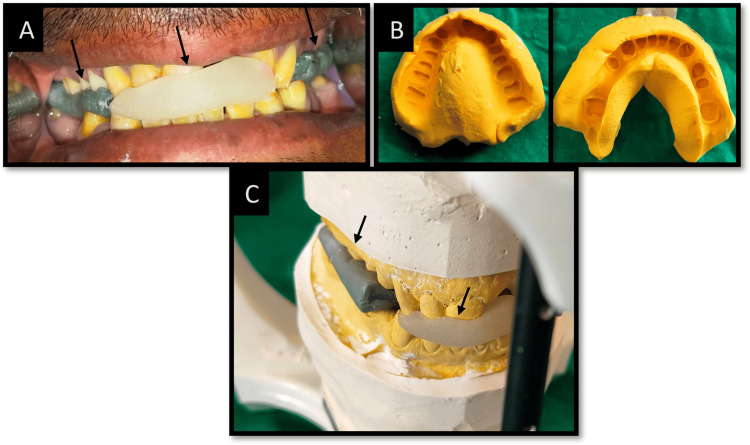
(A) Intraoral view showing the bite registration material used to record the established maxillomandibular relationship at the proposed vertical dimension. (B) Maxillary and mandibular alginate impressions for fabrication of provisional restorations. (C) Mounted casts on a semi-adjustable articulator demonstrating verification of the established vertical dimension of occlusion and occlusal relationships using a jig

**Figure 12:**
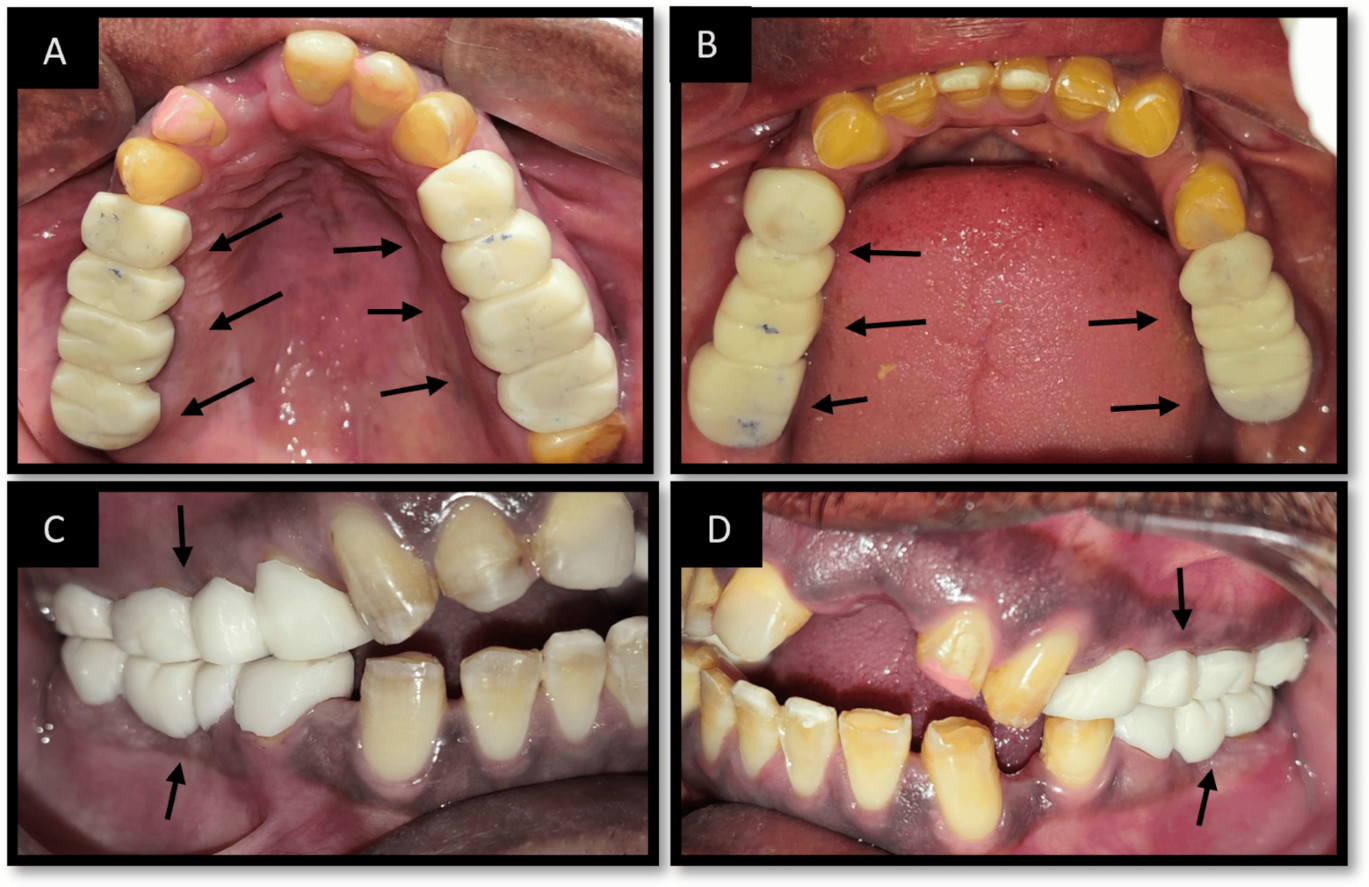
(A-D) Provisional restorations fabricated using a putty index derived from the diagnostic wax-up and temporarily cemented with zinc oxide non-eugenol cement to evaluate function, esthetics, and adaptation

**Figure 13:**
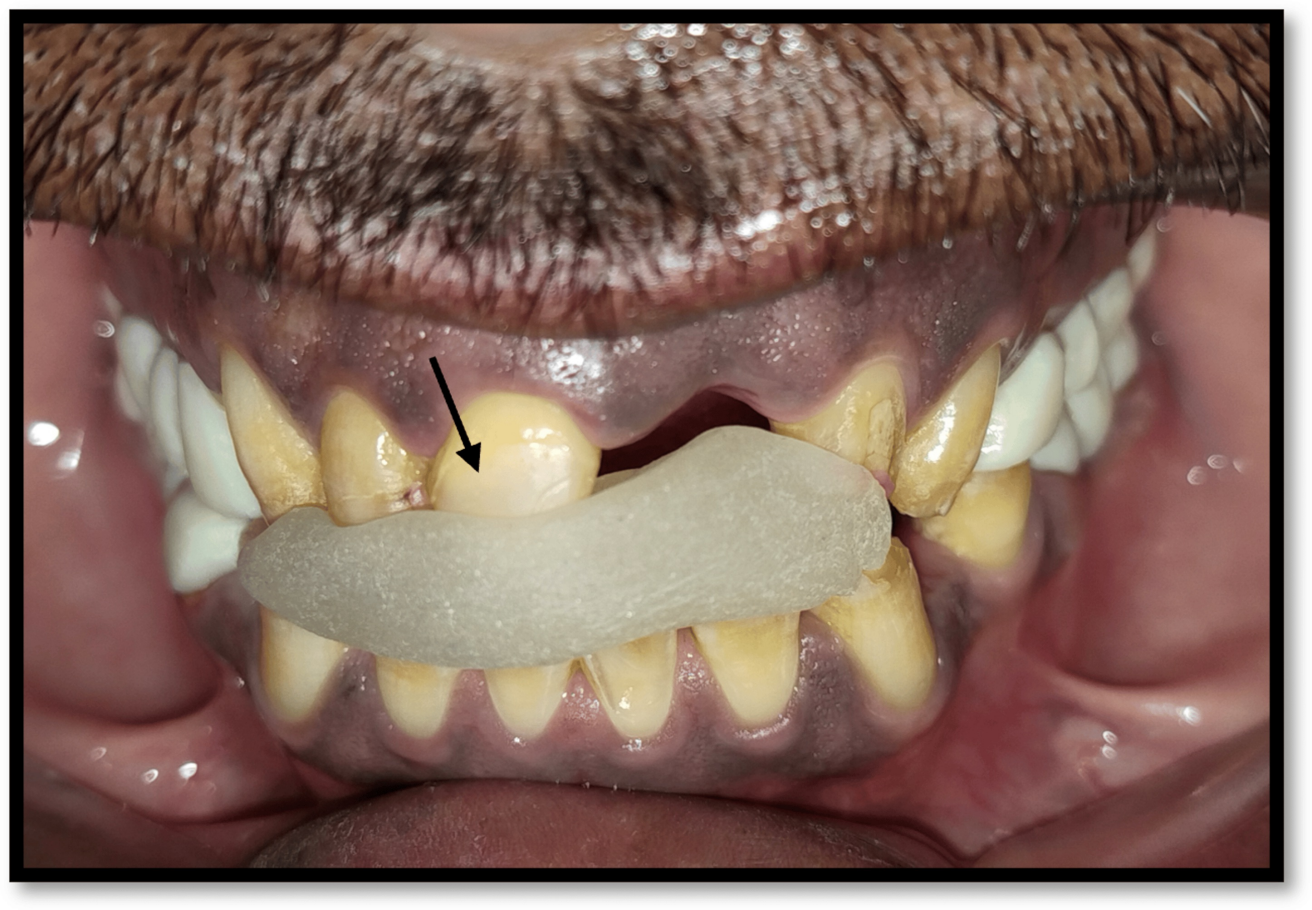
Intraoral view showing the use of a prefabricated jig to maintain the established vertical dimension of occlusion during provisionalization, ensuring consistency of occlusal relationships throughout the rehabilitation process

Posterior rehabilitation was initiated first to establish a stable occlusal foundation at the predetermined vertical dimension. Posterior teeth were prepared following biomechanical principles (Figures 10A, 10B) except for tooth 18, which was excluded from the treatment plan due to the absence of its antagonist (tooth 48), as it did not contribute to functional occlusion. Bite registration was performed to record the established maxillomandibular relationship (Figure 11A) followed by making alginate impressions (Figure 11B) for the fabrication of provisional crowns. The casts obtained were mounted on a semi-adjustable articulator, and the proposed VDO was carefully verified to ensure functional and esthetic harmony (Figure 11C). Provisional restorations were fabricated using a putty index derived from the diagnostic wax-up and temporarily cemented using zinc oxide non-eugenol cement (Figures 12A-12D). A prefabricated jig was used to maintain the established vertical dimension and centric relation during preparation and provisionalization (Figure 13).

**Figure 14:**
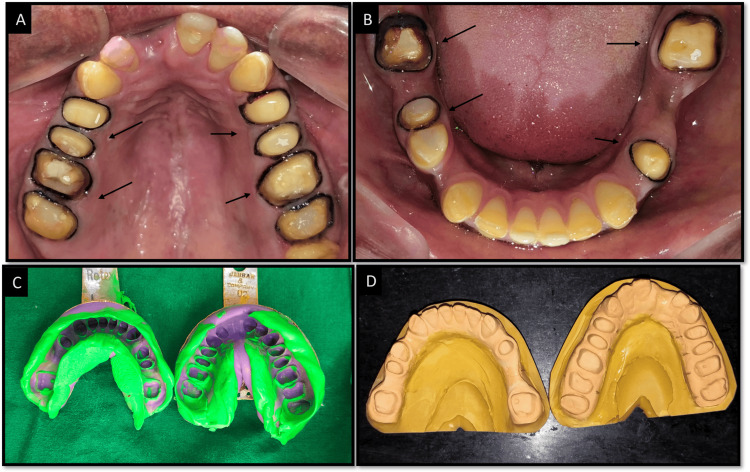
(A, B) Gingival retraction performed to expose the finish lines clearly, followed by (C, D) definitive impressions made using an elastomeric impression material to ensure accurate recording of prepared tooth margins and surrounding structures

The provisional restorations were maintained for a period of two weeks to evaluate the patient’s tolerance to the new mandibular position with the restored VDO followed by gingival retraction, and definitive impressions were made using an elastomeric impression material (Figures 14A-14D).

**Figure 15:**
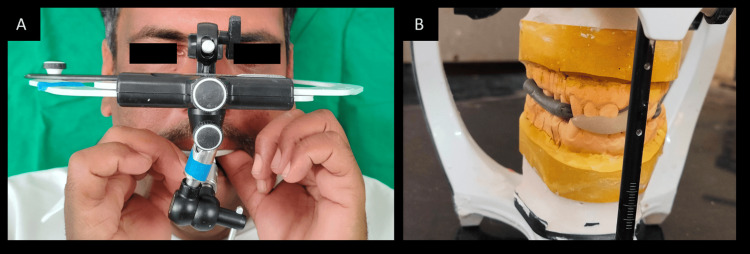
(A,B) Casts mounted on a semi-adjustable articulator using a facebow transfer and centric relation records, with verification of centric relation and vertical dimension performed using a Lucia jig

The casts were mounted using facebow transfer and centric relation records and verified with the Lucia jig (Figures 15A, 15B). Tooth 18 was extracted due to persistent pain and poor prognosis at this stage.

**Figure 16:**
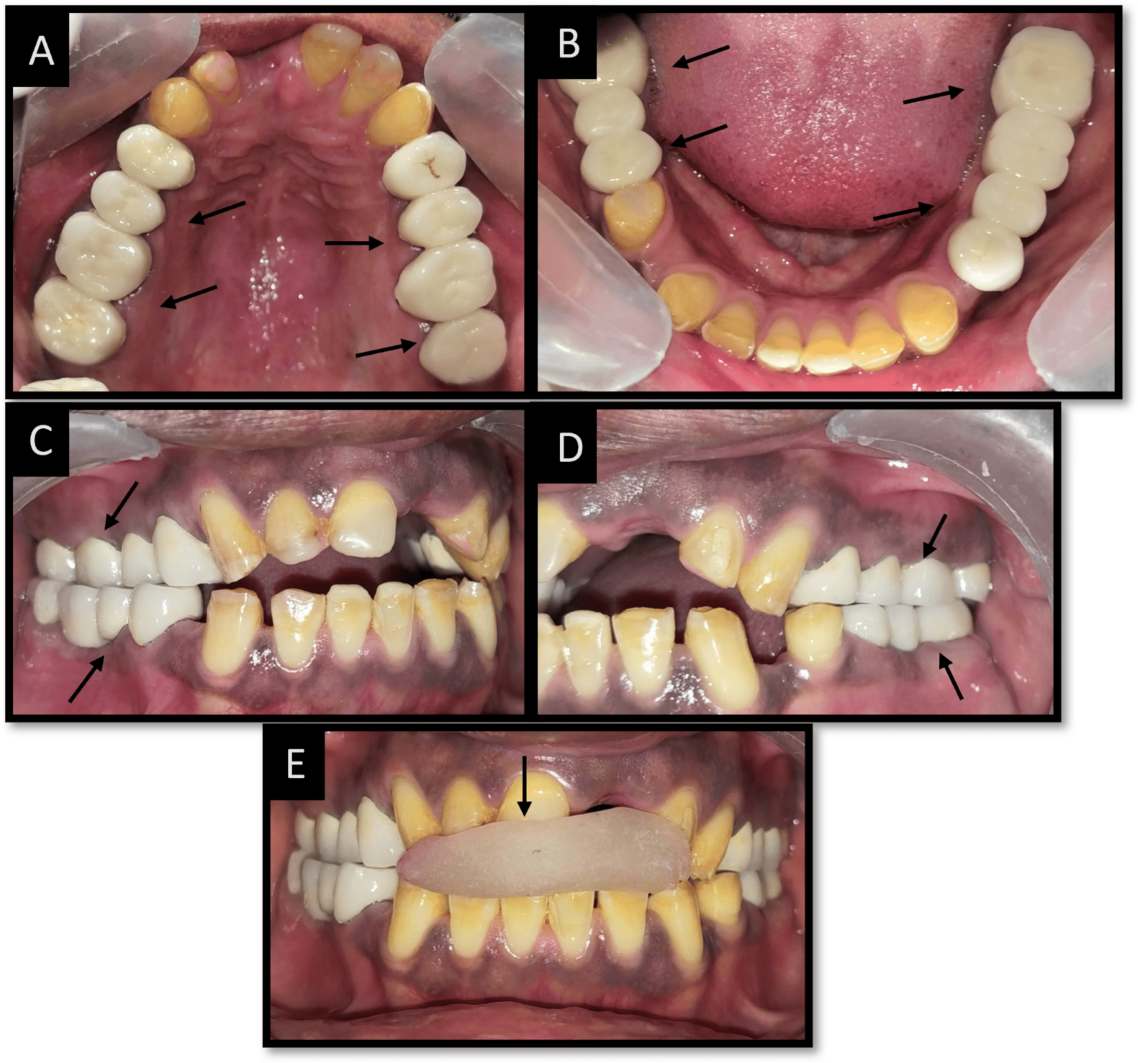
Posterior porcelain-fused-to-metal (PFM) restorations were cemented using glass ionomer cement. (A) Maxillary occlusal view, (B) mandibular occlusal view, (C) right lateral view, (D) left lateral view, and (E) frontal view; vertical dimension along with occlusion was clinically reverified

Metal coping trials were conducted to evaluate marginal fit and internal adaptation, followed by bisque trials to assess occlusion, proximal contacts, and morphology. After satisfactory evaluation, posterior PFM restorations were cemented using glass ionomer cement, and the vertical dimension and occlusion were reverified clinically (Figures 16A-16E).

**Figure 17:**
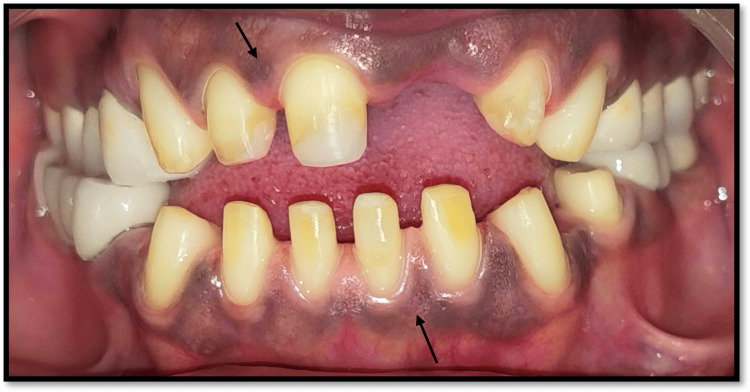
Frontal view showing anterior teeth preparation

Following stabilization of posterior occlusion, anterior rehabilitation was carried out. Anterior teeth were prepared conservatively to preserve tooth structure while ensuring adequate space for restorations (Figure 17).

**Figure 18:**
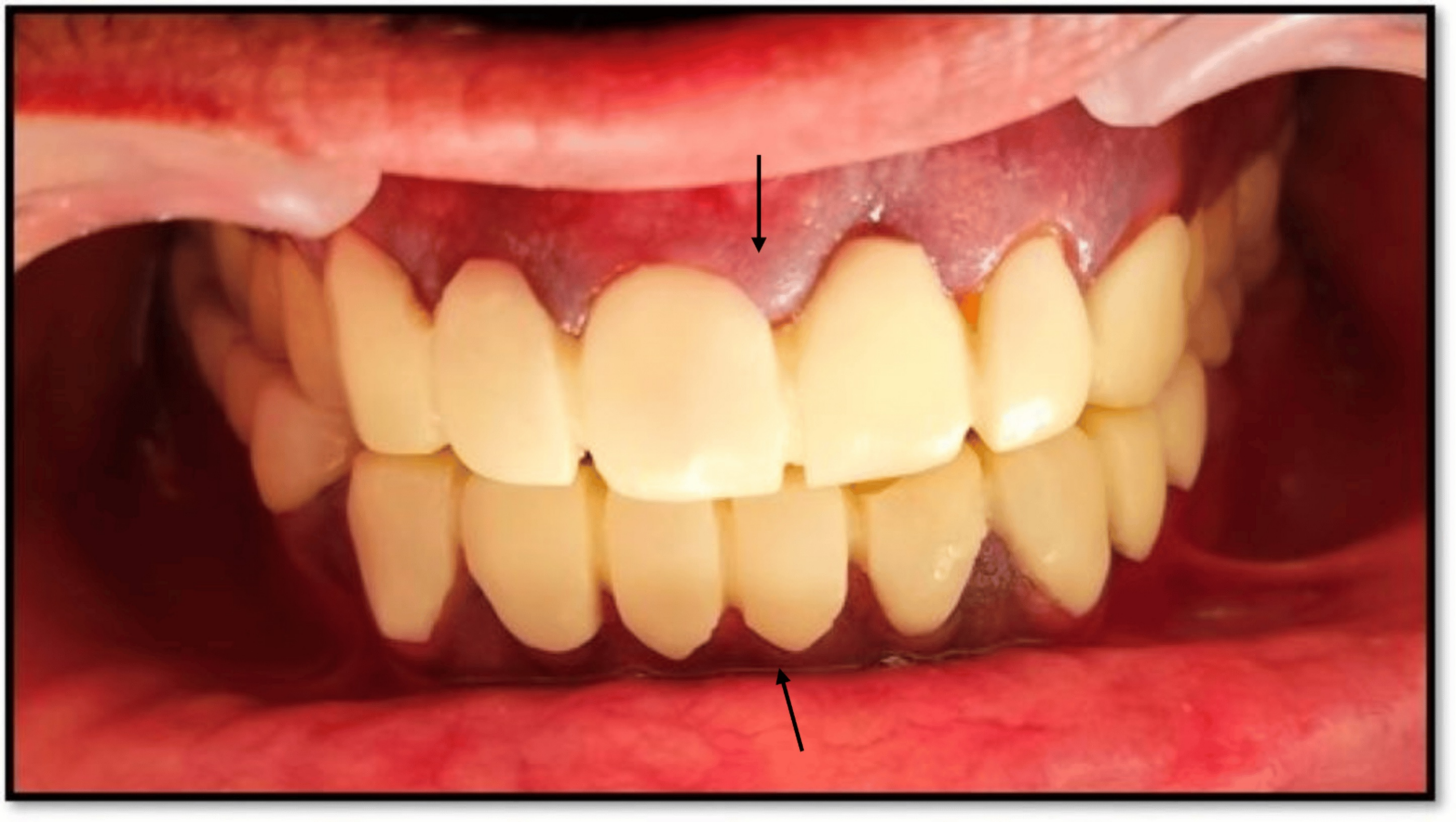
Anterior provisional restorations placed intraorally to evaluate esthetics, phonetics, and functional adaptation at the established vertical dimension of occlusion

Gingival retraction was performed, followed by elastomeric impressions and bite registration for the definitive anterior prosthesis, and subsequently, facebow recording and mounting were carried out. Anterior provisional restorations were placed to evaluate esthetics, phonetics, and functional adaptation (Figure 18).

The anterior open bite was corrected prosthetically by adjusting the vertical dimension and increasing incisal length to establish proper anterior contact. Phonetic evaluation using “F” and “V” sounds was performed during the temporization phase to determine the appropriate incisal edge position.

The unilateral crossbite was intentionally maintained to preserve the long axis of teeth and prevent excessive tooth reduction, thereby ensuring favorable biomechanical force distribution. Metal coping and bisque trials were performed to evaluate fit, esthetics, phonetics, and occlusion; upon satisfactory assessment, the definitive anterior restorations were fabricated and subsequently cemented.

**Figure 19:**
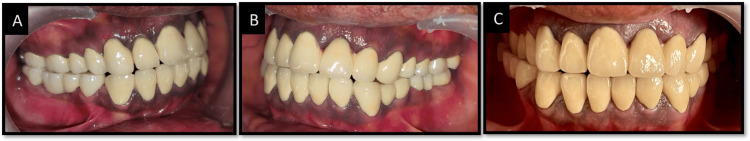
Definitive restorations in occlusion. (A) Right lateral view. (B) Left lateral view. (C) Frontal view illustrating the final esthetic outcome, incisal alignment, and smile harmony after definitive restoration placement

The final occlusal scheme was established as a group function occlusion to distribute occlusal forces across multiple teeth during lateral movements, thereby reducing stress on individual teeth. Occlusal equilibration was performed to eliminate interferences and ensure smooth mandibular movements. Post-treatment evaluation demonstrated improved function, esthetics, phonetics, and patient satisfaction, with no signs of temporomandibular discomfort (Figures 19A-19C).

## Discussion

Full-mouth rehabilitation is a complex and technique-sensitive procedure that requires meticulous planning, precise execution, and a thorough understanding of occlusal principles. It can be performed using either a simultaneous full-arch approach or a staged quadrant-wise approach, each having its own advantages and limitations. The simultaneous full-arch reconstruction technique allows complete control over occlusal plane development, esthetics, anterior guidance, and occlusal scheme, as all teeth are prepared and restored together. However, it is associated with increased chairside time, the need for full-arch anesthesia, greater technical complexity, and a higher risk of errors in recording and maintaining the VDO. In contrast, the quadrant-wise approach, as adopted in the present case, provides better clinical control, shorter appointments, and allows preservation of vertical dimension through the use of stable occlusal stops. Although it limits extensive occlusal modifications and may require careful coordination to maintain uniform esthetics, it offers a more predictable and patient-friendly treatment sequence [4,5].

The rehabilitation of patients with generalized enamel hypoplasia presents unique challenges due to the compromised quality and quantity of enamel. Enamel hypoplasia results in reduced structural integrity of teeth, making them more susceptible to wear, fracture, and sensitivity. In addition, long-standing enamel loss often leads to occlusal discrepancies, loss or alteration of vertical dimension, and functional disturbances. In the present case, these challenges were further compounded by the presence of an anterior open bite, unilateral crossbite, and multiple failing restorations. Such a clinical scenario necessitates a comprehensive prosthodontic approach that integrates biologic preservation, mechanical stability, and functional harmony [6,7].

One of the most critical aspects of full-mouth rehabilitation is the accurate determination of VDO. Any error in establishing VDO can lead to significant complications, including temporomandibular joint discomfort, muscle fatigue, impaired mastication, and failure of prosthetic restorations. Various methods have been described for determining VDO, including mechanical measurements, phonetic evaluation, esthetic assessment, and physiologic techniques. Among these, physiologic methods are considered more reliable because they are based on neuromuscular function rather than arbitrary measurements. In this case, the swallowing method was utilized as the primary means of determining VDO. During swallowing, mandibular positioning is guided by coordinated muscle activity and is considered a reproducible and physiologically balanced position. This makes it a dependable reference for establishing vertical dimension, especially in cases with altered occlusion [3,8,9].

To enhance the accuracy of centric relation recording and minimize proprioceptive interference from existing occlusal contacts, a Lucia jig was used. The use of such deprogramming devices is well documented in prosthodontic literature, as they help in achieving a stable and repeatable mandibular position. Additionally, the Shimbashi index was employed as an objective tool to verify the proposed vertical dimension, thereby combining both physiologic and metric approaches. This dual verification increased the reliability of the final VDO determination [10].

Another important consideration in this case was the sequencing of treatment. Conventionally, anterior guidance is often established first, but in this case posterior rehabilitation was carried out first as the posterior teeth were already prepared and lacked occlusal stops, establishing a new occlusal plane along with an appropriate occlusal vertical dimension was prioritized; therefore, posterior rehabilitation was carried out first to establish a stable occlusal platform at the corrected vertical dimension. This approach provided posterior support, facilitated accurate mandibular positioning, and created a foundation for subsequent anterior rehabilitation. Once posterior stability was achieved, anterior restorations were fabricated to establish proper guidance, phonetics, and esthetics.

Interim or provisional restorations play a crucial role in full-mouth rehabilitation. They serve not only as temporary replacements but also as diagnostic tools to evaluate the patient’s adaptation to changes in vertical dimension, occlusion, and esthetics. In the present case, provisional restorations allowed assessment of masticatory efficiency, speech, and comfort, and helped identify any potential issues before fabrication of definitive prostheses. The absence of temporomandibular discomfort or muscle fatigue during this phase indicated successful adaptation to the new vertical dimension [11].

The decision to maintain the unilateral crossbite rather than correcting it was based on sound biomechanical principles. Correction of crossbite would have required extensive tooth preparation, potentially compromising tooth structure and altering the long axis of the teeth. Maintaining the existing crossbite allowed preservation of tooth structure and ensured that occlusal forces were directed along the long axis, thereby enhancing the longevity of restorations. This highlights the importance of individualized treatment planning based on clinical findings rather than adhering strictly to ideal occlusal concepts [3,12,13].

The choice of porcelain-fused-to-metal restorations in this case was influenced by their proven clinical performance, strength, and durability. Patients with enamel hypoplasia often exhibit compromised tooth structure, necessitating the use of restorations that can withstand occlusal forces over a prolonged period. While all-ceramic restorations offer superior esthetics, PFM restorations provide a favorable balance between esthetics and mechanical strength, making them suitable for full-mouth rehabilitation cases involving high functional demands.

The final occlusal scheme adopted in this case was group function occlusion. In patients with weakened dentition or extensive restorations, group function is often preferred over canine guidance as it distributes occlusal forces across multiple teeth during lateral movements, thereby reducing stress on individual teeth. This approach enhances the longevity of restorations and minimizes the risk of fracture or failure.

Despite the successful outcome, certain limitations must be acknowledged. This report represents a single clinical case, and the results may not be universally applicable. Additionally, long-term follow-up was not included, which is essential to evaluate the durability and success of the treatment over time. Future studies involving larger sample sizes and long-term evaluation are necessary to validate the effectiveness of physiologic methods such as the swallowing technique in determining VDO in complex rehabilitative cases.

Overall, this case highlights the importance of a systematic and biologically driven approach in managing patients with generalized enamel hypoplasia and anterior open bite. Careful determination of vertical dimension, appropriate sequencing of treatment, use of provisional restorations, and adherence to sound prosthodontic principles are critical factors contributing to successful full-mouth rehabilitation.

## Conclusions

Full-mouth rehabilitation of patients with generalized enamel hypoplasia and anterior open bite requires careful diagnosis, accurate determination of the vertical dimension of occlusion, and systematic treatment planning. In this case, a physiologic approach using the swallowing technique, supported by the Lucia jig and Shimbashi index, facilitated predictable establishment of the vertical dimension and mandibular position.

A staged posterior-first rehabilitation approach and the use of provisional restorations aided in achieving functional adaptation and occlusal stability. This case highlights that a physiologically guided and individualized prosthodontic approach can successfully restore function, esthetics, and occlusal harmony in complex enamel hypoplasia cases. Further long-term studies are needed to support these findings.
